# Large-scale networks underlie cognitive insight differs between untreated adolescents ongoing their first schizophrenic episode and their reference non-schizophrenic mates

**DOI:** 10.1016/j.heliyon.2022.e10818

**Published:** 2022-09-30

**Authors:** Ruofei Ji, Ming Zhou, Na Ou, Hudan Chen, Yang Li, Lihua Zhuo, Xiaoqi Huang, Guoping Huang

**Affiliations:** a Department of Psychiatry, The Third Hospital of Mianyang, Mianyang, Sichuan, China; b Department of Radiology, The Third Hospital of Mianyang, Mianyang, Sichuan, China; c Department of Psychiatry, The Sixth Hospital of Nanchong, Nanchong, Sichuan, China; d Huaxi MR Research Center (HMRRC), Functional and Molecular Imaging Key Laboratory of Sichuan Province, Department of Radiology, West China Hospital, Sichuan University, Chengdu, Sichuan, China; e Department of Psychiatry, North Sichuan Medical College, Nanchong, Sichuan, China

**Keywords:** Adolescent-onset schizophrenia, Cognitive insight, Neuroimaging, Large-scale networks

## Abstract

**Objectives:**

Cognitive insight (CI), the ability to perceive erroneous beliefs and correcting them based on safe experiences, is a common cognitive manifestation among schizophrenic individuals. Even though the functional morphology of the default mode network (DMN), the central executive network (CEN) and the salience network (SN) differs between non-schizophrenic and schizophrenic individuals, it is unclear whether such differences are already in place by the first schizophrenic episode.

**Methods:**

Forty-two adolescents, including twenty-one AOS subjects was recruited, and performed independent component analysis (ICA) on resting-state fMRI data to explore alterations in the three networks in schizophrenia and the association of network changes with Beck Cognitive Insight Scale (BCIS) scores.

**Results:**

Compared to the non-schizophrenic group, the AOS group showed hyper-connectivity in the left middle temporal gyrus (MTG) and hypo-connectivity in the right parahippocampal gyrus within the DMN; hypo-connectivity in the dorsal anterior cingulate (dACC) and supplementary motor area (SMA) within the SN were also detected in AOS individuals. CI subscores were positively correlated with functional connectivity (FC) in the right parahippocampal gyrus.

**Conclusions:**

The correlations reported here suggest that increased DMN connectivity in the right parahippocampal gyrus might be an early neural correlate of reduced cognitive insight in a number, but not all, adolescent untreated individuals ongoing their first schizophrenic episode.

## 1 Introduction

The term schizophrenia encompasses a set of phenotypes whose lifetime prevalence reaches up to 0.6% in China (Huang et al., 2019), often characterized as dysfunction of cognitive, emotion and behaviour. Commonly, individuals ongoing their first schizophrenic episode during adolescence (AOS) display such event between 13 and 18 years of age. AOS individuals may exhibit genetic markers, disruptive social relationships, and poor responses to antipsychotics and psychotherapy. Lack of insight is a core manifestation of schizophrenia and indicates that individuals do not have a complete awareness of their psychotic disorder (Pick, 1882). With in-depth study, insight is more likely to be regarded as a neurocognition-involved issue. Beck and his colleagues proposed the concept of cognitive insight (CI), coupled with an additional cognitive dimension, defined as the ability to detect and correct one’s erroneous beliefs, and developed the Beck Cognitive Insight Scale (BCIS) to assess the capacity for revaluating anomalous experiences and misinterpretations (Beck et al., 2004). Compared with clinical insight, cognitive insight tends to be more stable and less impacted by the duration of illness, number of hospitalizations, age and years of education (Bora et al., 2007; Kim et al., 2015a).

Schizophrenia is thought to be the result of brain-wide functional dysconnectivity, and this hypothesis is supported by various neuroimaging evidence. Resting-state fMRI offers an approach to capture intrinsic spatiotemporal brain connectivity dynamics, which can reflect fundamental functional characteristics of the brain. The well-recognized resting-state networks mainly consist of the default mode network (DMN), executive control network (ECN), salience network (SN), dorsal attention network (DAN), and self-referential network (SRN). Dysfunction in one core network is often coupled with abnormalities in the other networks (Menon, 2011). The anomalous integration of information within and across these networks is important in linking the associated impaired cognitive features characteristic of psychopathology (Buckholtz and Meyer-Lindenberg, 2012; Gong et al., 2017). What is noteworthy is that aberrant resting state networks (RSNs) have a close relationship with self-related information processes (Camchong et al., 2011; Liu et al., 2012). Previous studies have claimed that cognitive insight is highly related to self-reflection, so we speculated that RSN dysfunction may cause cognitive insight disruption.

To further the understanding of how RSN function is relevant to phenotype with schizophrenia, the functional model was characterized in a development cohort. During this critical node of brain maturation progress, developmental delays get to crucial milestones, progressive grey matter volume loss and aberrant functional connectivity have been detected in early-onset case (Jacobsen and Rapoport, 1998; Tang et al., 2013), suggest a potentially deficit in information integrate and cognitive progress. Moreover, compared with adult-onset schizophrenia, early-onset schizophrenia appears to be more insidious (Kolvin et al., 1971; Eggers, 1978), often combine with premorbid maladjustment. The undiscovered cognitive impairment before illness onset might basis for decision making and self-judgment. Above all, we hypothesized a correlation between the alternative functional mode and the lack of cognitive insight in AOS.

Thus, in the present study, the authors aimed to explore the differences in network patterns in AOS individuals by using independent component analysis (ICA) with a main focus on the triple-network model. Furthermore, correlation analyses were applied to explore whether this is related to CI in AOS. It was hypothesized that AOS subjects may differ from non-schizophrenic in FC in the triple-network model, and those alterations may contribute to impaired CI.

## 22. Methods

### 2.1 Subjects

Twenty-one subjects with schizophrenia and 21 non-schizophrenic participated in this study. The following inclusion criteria for the subjects were applied: (1) they fulfilled the criteria of first-onset schizophrenia according to the Diagnostic and Statistical Manual of Mental Disorders, 5th edition (DSM-V); (2) they were 13–18 years of age and right handed; (3) illness duration was less than 1 year; (4) they had not received any medicine or other therapy; (5) there was no co-morbidity of Axis I disorders; (6) there was no history of major neurological or physical disorders; (7) there was no mental retardation; (8) there was no organic brain disorder and substance abuse; and (9) they were able to undergo MRI scans. The non-schizophrenic were recruited from school by advertisement and matched for age, sex and level of education. The study was performed in accordance with the Helsinki Declaration and approved by the Medical Ethics Committee of the Sichuan Mental Health Center. All the participants and their guardians signed the consent form after they were informed of the benefits and risks.

CI was measured by the BCIS(Beck et al., 2004). The BCIS is a 15-item self-rating scale that can be divided into two subscales according to the questions: 9 items for SR and 6 items for SC. The participants rated each item from 0 (do not agree at all) to 4 (agree completely) points, SR score minus SC score get composite index (range from −24 to 36), higher SR score and composite index reflect higher degree of CI. The Scale for the Assessment of Positive Symptoms (SAPS) and the Scale for the Assessment of Negative Symptoms (SANS) were applied to evaluate the severity of psychotic symptoms. In addition, we selected 11 items from the Psychotic Rating Scales (PSYRAT) to assess hallucinations (Drake et al., 2007), which investigated hallucinations from the following aspects:intensity, character, endurance and impact on life. All the scales except the BCIS were rated by an experienced doctor.

### 2.2 Data acquisition

All participants underwent a resting-state echo-planar imaging (EPI) scan through a 3.0T Siemens MRI scanner. They were informed to close their eyes, relax, stay awake, and avoid thinking and moving. Their heads were fixed by foam carpet to reduce head movement during the scan, and sound-insulated earphones were worn to prevent noise interference. Functional images were acquired with the following parameters: time points = 255, repetition time (TR) = 2000 ​m ​s, echo time (TE) = 30 ​m ​s, slice thickness = 4 mm, 35 axial slices, matrix:64 mm × 64 mm, flip angle: 90°, and field of view (FOV) = 240 mm × 240 mm. High-resolution magnetization-prepared fast gradient echo (MP-FGRE) T1-weighted images were also acquired (TR = 1900 ​m ​s, TE = 2.25 ​m ​s, slice thickness = 1 mm, matrix: 256 mm × 256 mm, and flip angle: 9°).

### 2.3 Image pre-processing

The functional images were preprocessed using the Data Processing Assistant for Resting-State fMRI (DPARSF version 4.4), which synthesizes procedures in statistical parametric mapping (SPM12, http://www.fil.ion.ucl.ac.uk/spm/software/spm12). The first 10 timepoints of the functional images were removed to ensure that all the images were acquired in a stable condition. The remaining 245 time points were corrected for slice-time differences and realigned to the first image. Structural images were co-registered to the mean functional image. The transformed structural images were then segmented into gray matter, white matter and cerebrospinal fluid (CSF). Then, mean time signals were extracted and regressed out. A higher-level Friston-24 model was used to regress head motion effects out of the realigned data (the 24 parameters included 6 head motion parameters, 6 head motion parameters one time point before, and the 12 corresponding squared items). The mean framewise displacement (FD) was further calculated as a measure of the microscale head motion of each subject. Data from subjects with high head movement (FD > 0.2 mm) were excluded. All images were spatially normalized into Montreal Neurological Institute (MNI) space (voxel size = 3 mm × 3 mm×3 mm). Finally, the images were smoothed with an 8-mm full-width half-maximum (FWHM) Gaussian kernel.

### 2.4 Independent component analysis

The ICA method was used for the preprocessed data with the Group ICA FMRI Toolbox (GIFT; http://icatb.sourceforge.net/gift/gift_startup.php).The individual components were estimated using the maximum description length (MDL) criterion. Data reduction was performed with a two-stage principal component analysis (PCA) that consisted of individual-level analysis and group-level analysis. After reduction, the infomax algorithm was used to decompose the data into a fixed set of 20 spatially independent components, and then the default method in the GIFT toolbox was used to test the stability of those components. Because the components we obtained from the above step belong to the group but not individuals, the data were back-reconstructed to obtain the spatial maps and time courses of individual components. The independent components were subsequently categorized into different intrinsic functional networks based on spatial correlation (*r* > 0.2) with a priori templates (Beckmann et al., 2005; Smith et al., 2009), and then visually confirmed the results. Finally, three intrinsic functional networks was identified with 6 independent components.

### 2.5 Statistical analyses

Spatial maps of selected components were compared between subjects and non-schizophrenic separately to acquire the components. Then, the common parts of the masks from the AOS group and non-schizophrenic group were used to create one mask for further group comparison.

To find the differences in spatial maps between the AOS and non-schizophrenic groups, components from both groups were entered in a two-sample t-test with the previously constructed mask. Next, the statistical significance of the results was defined using a threshold of p < 0.01 (uncorrected) at the voxel level and AlphaSim-corrected p < 0.05 at the cluster level. A functional network connectivity (FNC) correlation matrix was created to investigate FC between these three networks using the MANCOVA toolbox. Two-sample t-tests were applied to compare FNC correlations between the AOS and non-schizophrenic groups using false discovery rate (FDR) (q = 0.05) correction.

Demographic data, such as age, sex and years of education, were compared using t-tests or chi-square tests. Pearson correlation analyses were used between CI and clinical data, and FDR adjustment was applied (p < 0.05). Linear correlation analyses were used to explore the relationships between the FC of target brain areas and the clinical symptoms and CI.

## 3 Results

### 3.1 Demographics and clinical characteristics

**Figure 1:**
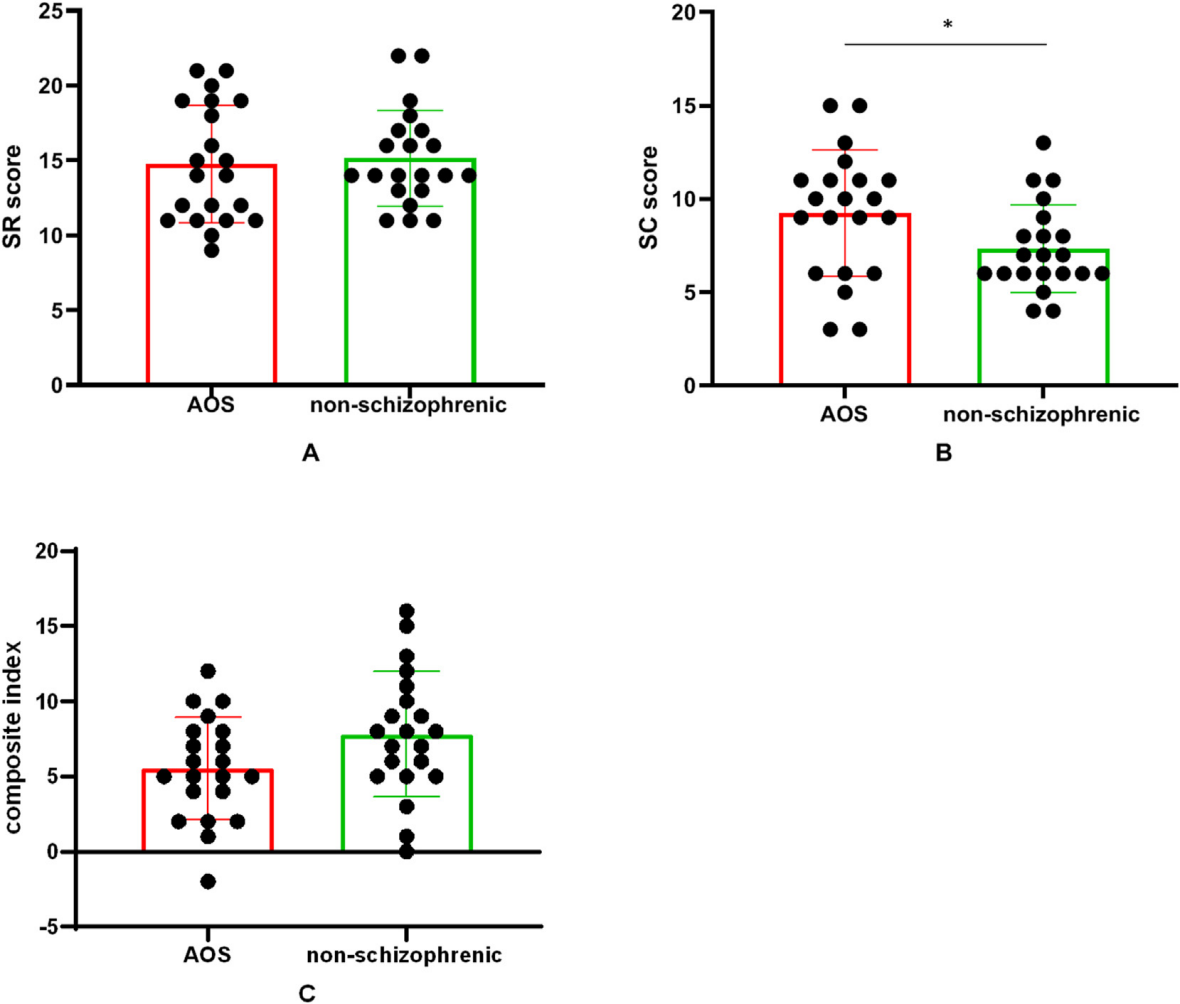
Compared with the non-schizophrenic group, the AOS group showed a significant increase in B) SC scores. No significant difference was found in A) SR scores or C) composite index. Abbreviations: AOS, adolescent-onset schizophrenia; SR, self-reflectiveness; SC, self-certainty.∗P < 0.05.

The AOS group and non-schizophrenic group did not have significant difference in age (*t* = -1.360,*p* = 0.182), sex ratio (*χ2* = 0.429,*p* = 0.513), or years of education (*t* = -1.210,*p* = 0.234). The mean SAPS, SANS and BCIS scores of these two groups are shown in Table 1. The AOS group showed significantly higher SC scores than the non-schizophrenic group (*t* = −2.116,*p* = 0.041) (Figure 1B), there was no significant difference in SR scores and composite index (Figure 1A, 1C). The Pearson correlation analysis between cognitive insight and clinical scale values showed that there were no significant result at the FDR corrected thresholds (Table 2).

**Table 1:** Clinical and demographic characteristics of AOS individuals and non-schizophrenic.

	HC group	AOS group	*t*/*χ*^*2*^	*p*
	(n = 21)	(n = 21)		
Age (years)	16.4 ± 1.0	16.9 ± 1.3	-1.360	0.182
Sex (male/female)	8/13	6/15	0.429	0.513
Years of education (years)	10.1 ± 1.3	10.6 ± 1.5	-1.21	0.234
BCIS				
Self-reflectiveness (SR)	15.1 ± 3.2	14.8 ± 3.9	0.345	0.732
Self-certainty (SC)	7.3 ± 2.4	9.2 ± 3.4	-2.116	0.041∗
Composite Index	7.8 ± 4.1	5.5 ± 3.4	1.954	0.058
SAPS	—	28.38 ± 13.02	—	—
SANS	—	33.57 ± 25.65	—	—
PSYRAT		18.10 ± 16.91		

SR, self-reflectiveness; SC, self-certainty; SAPS, Scale for Assessment of Positive Symptoms, SANS: Scale for Assessment of Negative Symptoms; PSYRAT, Psychotic Rating Scales.

∗ AOS > Controls (*p* < 0.05).

**Table 2:** Correlation analysis∗ between cognitive insight and clinical scales.

	SR	SC	composite index
Age (years) (P)	0.322 (0.385)	0.274 (0.491)	0.099 (0.675)
Years of education (years) (P)	0.417 (0.300)	0.343 (0.385)	0.139 (0.631)
SAPS (P)	-0.249 (0.519)	-0.097 (0.675)	-0.190 (0.558)
SANS (P)	-0.192 (0.558)	-0.471 (0.300)	0.249 (0.519)
PSYRAT (P)	0.423 (0.300)	0.162 (0.604)	0.327 (0.385)

SR, self-reflectiveness; SC, self-certainty; SAPS, Scale for Assessment of Positive Symptoms; SANS, Scale for Assessment of Negative Symptoms; PSYRAT, Psychotic Rating Scales.

∗ at the FDR correction threshold.

### 3.2 Within-network dysconnectivity

**Figure 2:**
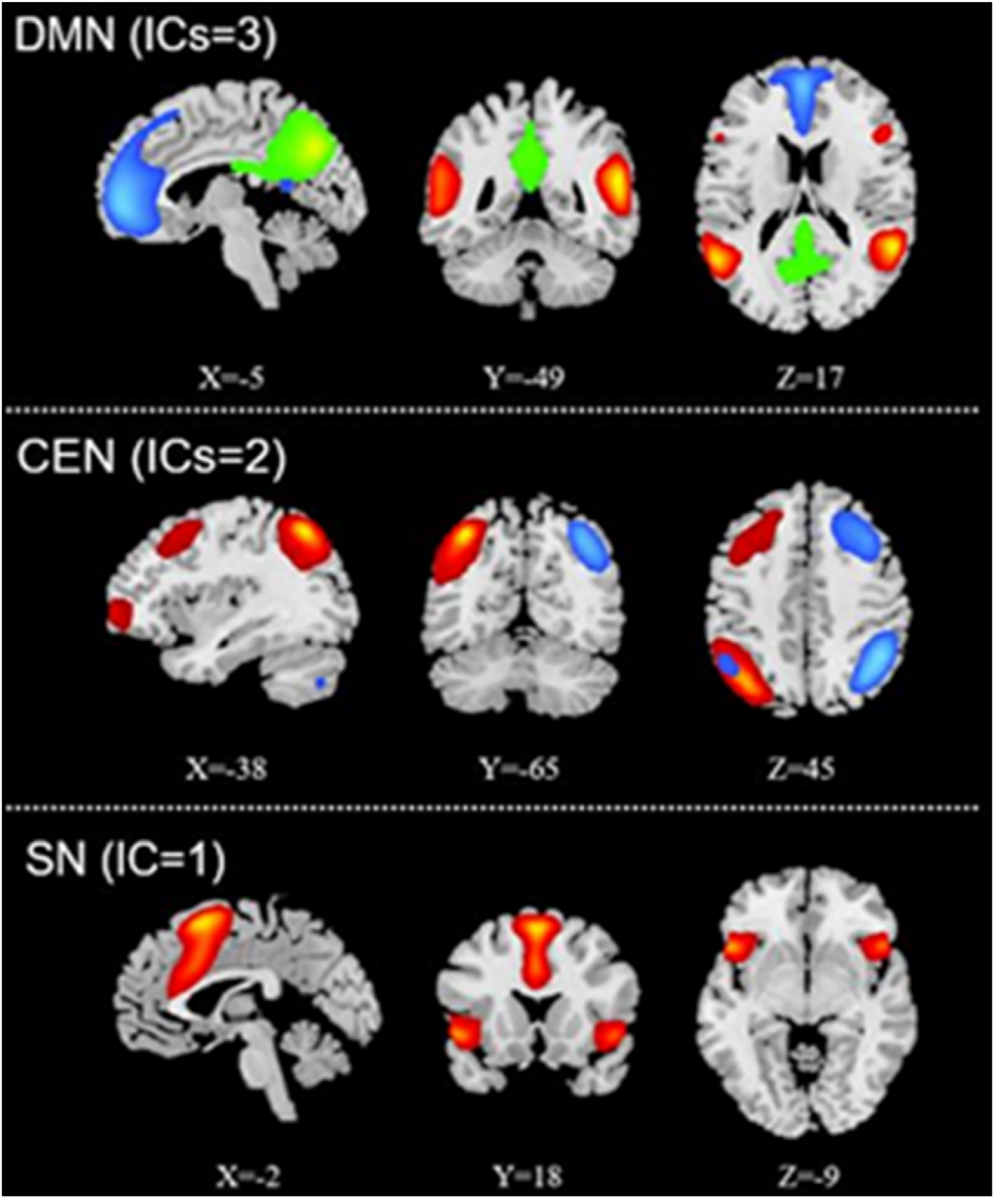
Three intrinsic functional networks extracted by group independent component analysis (ICA). Abbreviations: DMN, default mode network; CEN, central executive network; SN, salience network.

**Figure 3:**
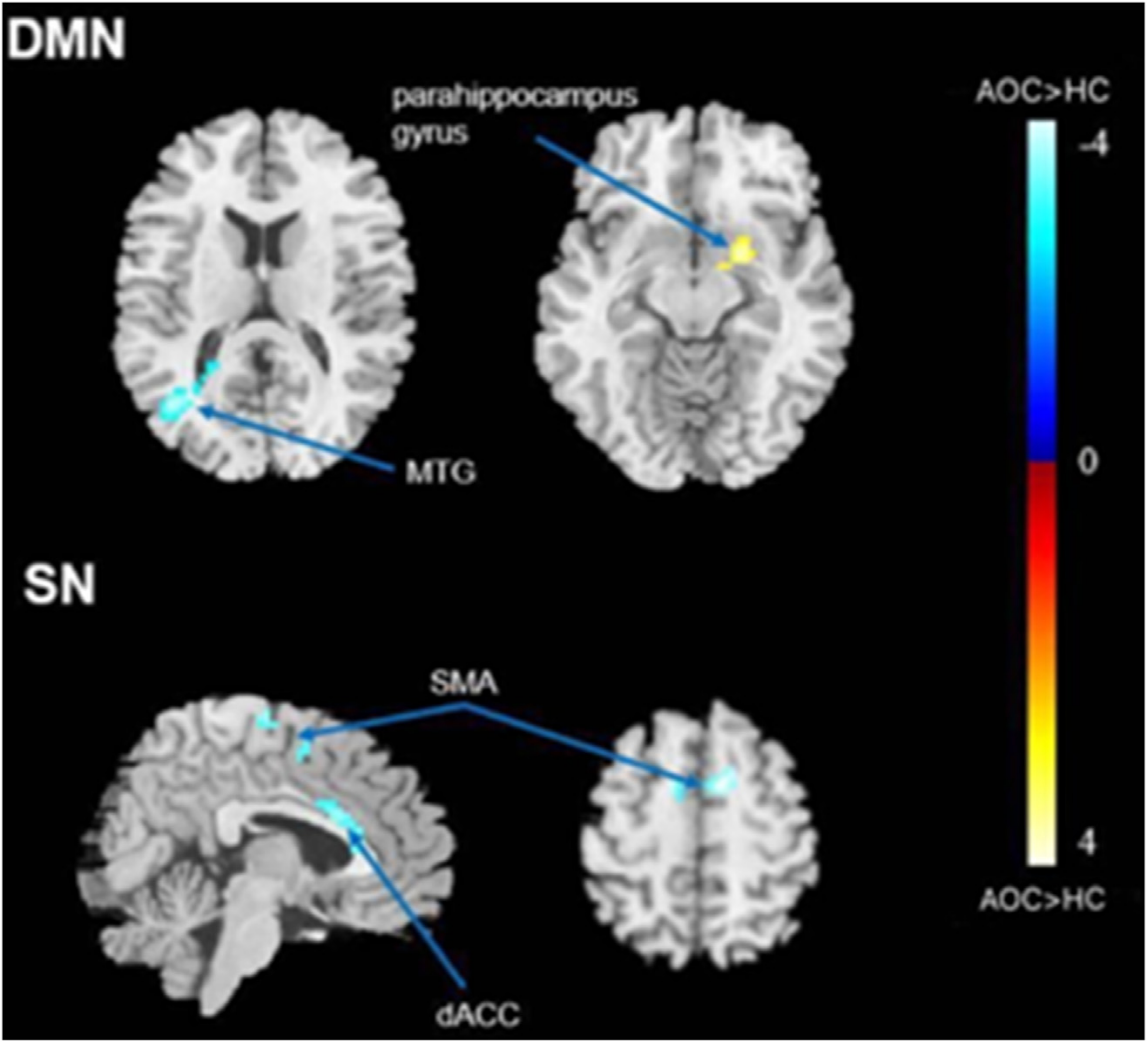
Differences between the AOS and non-schizophrenic groups in the within-network connections. Blue represents lower functional connectivity in the AOS subjects than in the non-schizophrenic. Yellow color represents higher functional connectivity in the AOS subjects than in the non-schizophrenic. Abbreviations: DMN, default mode network; SN, salience network. MTG, middle temporal gyrus; dACC, dorsal anterior cingulate cortex; SMA, supplementary motor area.

**Figure 4:**
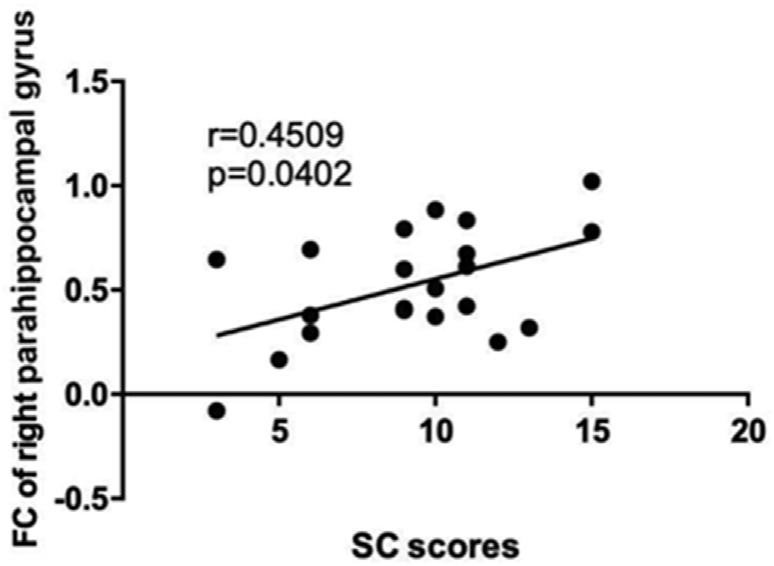
Functional connectivity strength within the right parahippocampal gyrus was positively correlated with SC scores in the AOS group. Abbreviations: AOS, adolescent-onset schizophrenia; SC, self-certainty.

The intrinsic functional networks extracted by group ICA are presented in Figure 2 Compared to the non-schizophrenic group, the AOS group showed imbalanced FC within the DMN with weaker connectivity at the level of the left middle temporal gyrus (MTG) but stronger connectivity for the right parahippocampal gyrus. In addition, the AOS group had weaker FC within the SN in the dorsal anterior cingulate cortex (dACC) and supplementary motor area (SMA) (voxel-level *p* < 0.01,cluster-level *p* < 0.05, AlphaSim correction) (Figure 3, Table 3). No significant results were found within the CEN between these two groups. Further analysis showed that the SC scores were positively related to the FC strength of the right parahippocampal gyrus (*r* = 0.451, *p* < 0.05) (Figure 4).

**Table 3:** The significant brain areas between the AOS and non-schizophrenic groups within the DMN and SN.

Cluster location	Cluster size	Peak MNI			t value	P value (uncorrected)
		x	y	z		
DMN						
Patients < Controls						
Left MTG	197	-36	-69	21	-4.41	<0.001
Patients > Controls						
Right parahippocampal gyrus	91	21	3	-12	3.96	0.009
SN						
Patients < Controls						
dACC	146	-9	9	30	-3.76	0.001
SMA	125	9	3	57	-3.77	0.003

MTG, middle temporal gyrus; dACC, dorsal anterior cingulate; SMA, supplementary motor area.

∗Results are reported for voxel-level p < 0.01 and AlphaSim correction.

### 3.3 Between-network dysconnectivity

There were no significant differences in between-network connectivity between the AOS and non-schizophrenic groups.

## 4 Discussion

Schizophrenia is defined as a kind of severe mental disorder characterized by deficit in the relation between thought, emotion, and behaviour, often accompany with impaired cognitive insight. As expected, AOS demonstrated alternative functional connectivity in intrinsic neural network, and this differs were associated with cognitive insight. This results indicated that the alternative functioning of the right parahippocampal gyrus within the DMN probably lead to improper self-referential information process, might be the potential foundation of CI impairments. To our knowledge, no previous work have evaluated whether reduced cognitive insight is already present in adolescent untreated individuals ongoing their first schizophrenic episode.

The only study exploring the relationship between CI and RSN found that CI was associated with functional connectivity in the right inferior frontal cortex (IFC) and left ACC in adult schizophrenia subjects (Gerretsen et al., 2014). This study presented hyper-connectivity in the right parahippocampal gyrus within the DMN in AOS individuals, that increased connectivity in the AOS subjects compared to the non-schizophrenic was related to higher SC scores. Authors have suggested that the parahippocampal gyrus is engaged extensively in cognitive processing such as memory recollection (Gary and Van, 1982; Eichenbaum et al., 1996), hallucinations (Diederen et al., 2010), and emotion (Arias et al., 2015; Kim et al., 2015b). Besides, the medial temporal subsystem in DMN, including the parahippocampal gyrus, has been implicated in past and future autobiographical thought and contextual retrieval (Andrews-Hanna et al., 2014). One study with self-judgment reported co-activation between the VMPFC and parahippocampus gyrus while making self-judgments, prompting the retrieval of specific autobiographical details to influence self-referential information assessments while making self-judgments (Feyers et al., 2010). This research was predicated on the theory that RSNs dysfunction contribute to deficit self-related information processes. According to the description, the SC score refers to self-evaluation and self-judgment, which fall largely in the domain of self-reference. As mentioned before, parahippocampal gyrus plays a critical role in autobiographical memory and contextual information processing, it can be inferred that the parahippocampal gyrus participates in self-judgment through its engagement in autobiographical memory, which could explain the positive correlation that found between SC scores and FC in the parahippocampal gyrus. This work employed first-onset drug-naïve AOS individuals and provides evidence that increased DMN connectivity in the right parahippocampal gyrus might be an indication of the reduction of cognitive insight in individuals during development.

The DMN is well known for its involvement in self-referential (Gusnard et al., 2001), integrative processes (Greicius et al., 2003), internal emotion (Raichle and Snyder, 2007) and internal thought (Mason et al., 2007). In the current study, the dysconnectivity in the left MTG within DMN are consistent with previous resting-state studies with AOS subjects (Zhang et al., 2017). As an important region of the DMN, abundant evidences has emphasized that the MTG is involved in important cognitive processes (Cabeza and Nyberg, 2000; Kristinsson et al., 2020) and plays an influential role in the activation of psychiatric symptoms (Zhang et al., 2017; Cui et al., 2018). Otherwise, the MTG has been suggested to be crucial in self-representations by retrieval information from autobiographical memory (Feng et al., 2018). On the other hand, AOS subjects presented significant hypo-connectivity in the dACC and SMA within the SN. These results are in line with previous studies with schizophrenia (Pu et al., 2012), while no relevant study with AOS subject can be identified. The SN detects the salience of internal and external stimuli (Sridharan et al., 2008; Menon and Uddin, 2010), alternative attribution of salience to external and internal stimuli is thought to arouse psychotic symptoms. For instance, Mallikarjun et al. (2018) reported dysconnectivity between the SN and DMN in first-episode schizophrenia with auditory verbal hallucinations, similar results also appeared in chronic schizophrenia (Alonso-Solis et al., 2015) and individuals with PTSD (Cheng et al., 2019). Moreover, variety in interactions between the SN and prefrontal system, which may lead to an unbalanced salience-execution loop, have been hypothesized to underlie the neural background of psychosis (Palaniyappan et al., 2013). In addition, alternative integration between the SN and DMN and the CEN in schizophrenia have already reported (Wu and Jiang, 2019; Manoliu et al., 2014), implied that the ability to coordinate self-monitoring and task performance was impaired in schizophrenia individuals; thus, the reduced requirement of the ACC and SMA suggest a disturbance in this system.

This research failed to find a significant difference in the CEN between AOS individuals and non-schizophrenic. The CEN is mainly involved in working memory and decision making (Michael, 2005; Koechlin and Summerfield, 2007), dysfunction in the CEN has been widely reported in schizophrenia, and both hyper-connectivity (Krishnadas et al., 2014) and hypo-connectivity (Woodward et al., 2011) have been found. However, parallel with the development of the neural system, brain networks are also altered during adolescence (Menon, 2013). Thus, AOS allows researchers to treat schizophrenia as alternative phenotypes of brain connectivity emerging from divergent neurodevelopmental trajectories. Based on the small sample size, it is hard to support the hypothesis of CEN dysfunction in AOS, amplifying the quantity of subjects may have a different result.

Although triple-network model alterations was examined on a large scale with reliable measurements in an adolescent population with schizophrenia, the present study still had several limitations. First, restricting the sample to drug-naïve adolescents enhanced the homogeneity but limited the generalizability of our results to adult individuals. Second, the sample size was relatively small, and future fMRI studies in a larger sample will be needed to validate these findings. Last, the conclusion is based on adolescent-onset untreated individuals ongoing their first schizophrenic episode, for the reason that schizophrenia might be a continuum of phenotypes, only in a certain percentage increased DMN connectivity in the right parahippocampal gyrus could be treated as early neural marker of impaired cognitive insight.

In conclusion, we characterized the large-scale network changes underlying CI in untreated adolescents ongoing their first schizophrenic episode. In line with previous predictions, this results showed that dysfunction in the right parahippocampal gyrus within the DMN might be an early neural correlate of reduced cognitive insight, provide neurobiological evidence to support the theory of brain-behaviors relationships is critical for cognitive process and clinical symptoms, offer fundamental information for further research

## Declarations

### Author contribution statement

Guoping Huang; Xiaoqi Huang: Conceived and designed the experiments; Wrote the paper.

Ruofei Ji; Ming Zhou: Performed the experiments; Analyzed and interpreted the data; Wrote the paper.

Na Ou; Hudan Chen; Yang Li: Contributed reagents, materials, analysis tools or data; Wrote the paper.

### Funding statement

Dr. Guoping Huang was supported by National Key Research and Development program of China [2016YFC1307000].

### Data availability statement

The authors do not have permission to share data.

### Declaration of interest’s statement

The authors declare no conflict of interest.

### Additional information

No additional information is available for this paper.

## References

[bib1] Alonso-Solis A., Vives-Gilabert Y., Grasa E., Portella M.J., Rabella M., Sauras R.B., Roldan A., Nunez-Marin F., Gomez-Anson B., Perez V., Alvarez E., Corripio I. (2015). Resting-state functional connectivity alterations in the default network of schizophrenia patients with persistent auditory verbal hallucinations. Schizophr. Res..

[bib2] Andrews-Hanna J.R., Smallwood J., Spreng R.N. (2014). The default network and self-generated thought: component processes, dynamic control, and clinical relevance. Ann. N. Y. Acad. Sci..

[bib3] Arias N., Mendez M., Arias J.L. (2015). The importance of the context in the hippocampus and brain related areas throughout the performance of a fear conditioning task. Hippocampus.

[bib4] Beck A.T., Baruch E., Balter J.M., Steer R.A., Warman D.M. (2004). A new instrument for measuring insight: the Beck Cognitive Insight Scale. Schizophr. Res..

[bib52] Beckmann C.F., DeLuca M., Devlin J.T., Smith S.M. (2005). Investigations into resting-state connectivity using independent component analysis. Philos. Trans. R. Soc. Lond. B Biol. Sci..

[bib6] Buckholtz J.W., Meyer-Lindenberg A. (2012). Psychopathology and the human connectome: toward a transdiagnostic model of risk for mental illness. Neuron.

[bib7] Cabeza R., Nyberg L. (2000). Imaging cognition II: an empirical review of 275 PET and fMRI studies. J. Cognit. Neurosci..

[bib8] Camchong J., MacDonald AW B.C., Mueller B.A., Lim K.O. (2011). Altered functional and anatomical connectivity in schizophrenia. Schizophr. Bull..

[bib9] Cheng L.L., Zhu J.J., Ji F., Lin X.D., Zheng L.D., Chen C., Chen G.D., Xie Z.L., Xu Z.J., Zhou C.H., Xu Y., Zhuo C.J. (2019). Add-on atypical anti-psychotic treatment alleviates auditory verbal hallucinations in patients with chronic post-traumatic stress disorder. Neurosci. Lett..

[bib11] Cui Y., Liu B., Song M., Lipnicki D.M., Li J., Xie S., Chen Y., Li P., Lu L., Lv L., Wang H., Yan H., Yan J., Zhang H., Zhang D., Jiang T. (2018). Auditory verbal hallucinations are related to cortical thinning in the left middle temporal gyrus of patients with schizophrenia. Psychol. Med..

[bib50] Diederen K.M., Neggers S.F., Daalman K., Blom J.D., Goekoop R., Kahn R.S., Sommer I.E. (2010). Deactivation of the parahippocampal gyrus preceding auditory hallucinations in schizophrenia. Am. J. Psychiatry.

[bib12] Drake R., Haddock G., Tarrier N., Bentall R., Lewis S. (2007). The Psychotic Symptom Rating Scales (PSYRATS): their usefulness and properties in first episode psychosis. Schizophr. Res..

[bib13] Eggers C. (1978). Course and prognosis in childhood schizophrenia. J. Autism Child. Schizophr..

[bib14] Eichenbaum H., Schoenbaum G., Young B., Bunsey M. (1996). Functional organization of the hippocampal memory system. Proc. Natl. Acad. Sci. U. S. A.

[bib15] Feng C., Yan X., Huang W., Han S., Ma Y. (2018). Neural representations of the multidimensional self in the cortical midline structures. Neuroimage.

[bib16] Feyers D., Collette F., D'Argembeau A., Majerus S., Salmon E. (2010). Neural networks involved in self-judgement in young and elderly adults. Neuroimage.

[bib18] Gerretsen P., Menon M., Mamo D.C., Fervaha G., Remington G., Pollock B.G., Graff-Guerrero A. (2014). Impaired insight into illness and cognitive insight in schizophrenia spectrum disorders: resting state functional connectivity. Schizophr. Res..

[bib19] Gong Q., Hu X., Pettersson-Yeo W., Xu X., Lui S., Crossley N., Wu M., Zhu H., Mechelli A. (2017). Network-level dysconnectivity in drug-naive first-episode psychosis: dissociating transdiagnostic and diagnosis-specific alterations. Neuropsychopharmacology.

[bib20] Greicius M.D., Krasnow B., Reiss A.L., Menon V. (2003). Functional connectivity in the resting brain: a network analysis of the default mode hypothesis. Proc. Natl. Acad. Sci. U. S. A.

[bib21] Gusnard D.A., Akbudak E., Shulman G.L., Raichle M.E. (2001). Medial prefrontal cortex and self-referential mental activity: relation to a default mode of brain function. Proc. Natl. Acad. Sci. U. S. A.

[bib23] Huang Y., Wang Y., Wang H., Liu Z., Yu X., Yan J., Yu Y., Kou C., Xu X., Lu J., Wang Z., He S., Xu Y., He Y., Li T., Guo W., Tian H., Xu G., Xu X., Ma Y., Wang L., Wang L., Yan Y., Wang B., Xiao S., Zhou L., Li L., Tan L., Zhang T., Ma C., Li Q., Ding H., Geng H., Jia F., Shi J., Wang S., Zhang N., Du X., Du X., Wu Y. (2019). Prevalence of mental disorders in China: a cross-sectional epidemiological study. Lancet Psychiatr..

[bib24] Jacobsen L.K., Rapoport J.L. (1998). Research update: childhood onset schizophrenia: implications of clinical and neurobiological research. J. Child. Psychol. Psychiatry.

[bib25] Kim G.W., Yang J.C., Jeong G.W. (2015). Emotional effect on cognitive control in implicit memory tasks in patients with schizophrenia. Neuroreport.

[bib26] Kim J.H., Lee S., Han A.Y., Kim K., Lee J.Y. (2015). Relationship between cognitive insight and subjective quality of life in outpatients with schizophrenia. Neuropsychiatric Dis. Treat..

[bib27] Koechlin E., Summerfield C. (2007). An information theoretical approach to prefrontal executive function. Trends Cognit. Sci..

[bib28] Kolvin I., Ounsted C., Humphrey M., McNay A. (1971). Studies in the childhood psychoses. II. The phenomenology of childhood psychoses. Br. J. Psychiatry.

[bib29] Krishnadas R., Ryali S., Chen T., Uddin L., Supekar K., Palaniyappan L., Menon V. (2014). Resting state functional hyperconnectivity within a triple network model in paranoid schizophrenia. Lancet.

[bib30] Kristinsson S., Thors H., Yourganov G., Magnusdottir S., Hjaltason H., Stark B.C., Basilakos A., den Ouden D.B., Bonilha L., Rorden C., Hickok G., Hillis A., Fridriksson J. (2020). Brain damage associated with impaired sentence processing in acute aphasia. J. Cognit. Neurosci..

[bib31] Liu H., Kaneko Y., Ouyang X., Li L., Hao Y., Chen E.Y., Jiang T., Zhou Y., Liu Z. (2012). Schizophrenic patients and their unaffected siblings share increased resting-state connectivity in the task-negative network but not its anticorrelated task-positive network. Schizophr. Bull..

[bib33] Mallikarjun P.K., Lalousis P.A., Dunne T.F., Heinze K., Reniers R.L., Broome M.R., Farmah B., Oyebode F., Wood S.J., Upthegrove R. (2018). Aberrant salience network functional connectivity in auditory verbal hallucinations: a first episode psychosis sample. Transl. Psychiatry.

[bib34] Manoliu A., Riedl V., Zherdin A., Muhlau M., Schwerthoffer D., Scherr M., Peters H., Zimmer C., Forstl H., Bauml J., Wohlschlager A.M., Sorg C. (2014). Aberrant dependence of default mode/central executive network interactions on anterior insular salience network activity in schizophrenia. Schizophr. Bull..

[bib35] Mason M.F., Norton M.I., Van Horn J.D., Wegner D.M., Grafton S.T., Macrae C.N. (2007). Wandering minds: the default network and stimulus-independent thought. Science.

[bib36] Menon V. (2011). Large-scale brain networks and psychopathology: a unifying triple network model. Trends Cognit. Sci..

[bib37] Menon V. (2013). Developmental pathways to functional brain networks: emerging principles. Trends Cognit. Sci..

[bib38] Menon V., Uddin L.Q. (2010). Saliency, switching, attention and control: a network model of insula function. Brain Struct. Funct..

[bib53] Michael P. (2005). Lateral prefrontal cortex: architectonic and functional organization. Philos. Trans. R. Soc. Lond. B Biol. Sci..

[bib39] Palaniyappan L., Simmonite M., White T.P., Liddle E.B., Liddle P.F. (2013). Neural primacy of the salience processing system in schizophrenia. Neuron.

[bib41] Pick A. (1882). Ueber Krankheitsbewusstsein in psychischen Krankheiten. Arch. Psychiatr. Nervenkr..

[bib42] Pu W., Li L., Zhang H., Ouyang X., Liu H., Zhao J., Li L., Xue Z., Xu K., Tang H., Shan B., Liu Z., Wang F. (2012). Morphological and functional abnormalities of salience network in the early-stage of paranoid schizophrenia. Schizophr. Res..

[bib43] Raichle M.E., Snyder A.Z. (2007). A default mode of brain function: a brief history of an evolving idea. Neuroimage.

[bib44] Smith S.M., Fox P.T., Miller K.L., Glahn D.C., Fox P.M., Mackay C.E., Filippini N., Watkins K.E., Toro R., Laird A.R., Beckmann C.F. (2009). Correspondence of the brain’s functional architecture during activation and rest. Proc. Natl. Acad. Sci. U. S. A.

[bib45] Sridharan D., Levitin D.J., Menon V. (2008). A critical role for the right fronto-insular cortex in switching between central-executive and default-mode networks. Proc. Natl. Acad. Sci. U. S. A.

[bib46] Tang J., Liao Y., Song M., Gao J.H., Zhou B., Tan C., Liu T., Tang Y., Chen J., Chen X. (2013). Aberrant default mode functional connectivity in early onset schizophrenia. PLoS One.

[bib48] Woodward N.D., Rogers B., Heckers S. (2011). Functional resting-state networks are differentially affected in schizophrenia. Schizophr. Res..

[bib51] Wu D., Jiang T. (2019). Schizophrenia-related abnormalities in the triple network: a meta-analysis of working memory studies. Brain Imaging Behav..

[bib49] Zhang L., Li B., Wang H., Li L., Liao Q., Liu Y., Bao X., Liu W., Yin H., Lu H., Tan Q. (2017). Decreased middle temporal gyrus connectivity in the language network in schizophrenia patients with auditory verbal hallucinations. Neurosci. Lett..

